# In situ antioxidant activity of a dermo‐cosmetic product: A randomized controlled clinical study

**DOI:** 10.1111/exd.14005

**Published:** 2019-09-30

**Authors:** Virginie Ribet, Vincenzo Nobile, Ana Beatris Rossi

**Affiliations:** ^1^ Clinical Skin Research Center Pierre Fabre Dermo‐Cosmetics Toulouse France; ^2^ Complife Italia San Martino Siccomario (PV) Italy

**Keywords:** lipid peroxidation, oxidative stress, photoageing, topical antioxidant, UVA

## Abstract

Ultraviolet light enhances the generation of reactive oxygen species that are responsible for skin photoageing. The aim of this randomized, vehicle‐ and active‐controlled double‐blind, intra‐individual monocentric study was to evaluate in situ the antioxidant activity of a dermo‐cosmetic product in photoaged skin. Twenty healthy volunteers had defined skin areas randomized to receive a topical product containing 3 antioxidants (pre‐tocopheryl^®^, retinaldehyde and glycylglycine ole‐amide), its vehicle and a positive antioxidant control cream. The products were applied daily for 30‐day period. The skin areas were exposed to a controlled dose of UVA rays, and the skin oxidative status was evaluated 4 and 24 hours post‐UVA exposure at D0 (basal value) and after 15 and 30 days of product application. Skin layers were collected by stripping, and antioxidant capacity was measured using the ferric reducing ability of a plasma assay. Lipid peroxidation (LPO) was assessed using the malonyldialdehyde test. The tested product significantly improved the skin antioxidant capacity after 15 and 30 days and significantly decreased the basal level of the skin LPO. The skin LPO level significantly decreased 4 and 24 hours after UVA exposure at 15 and 30 days. These findings were comparable to positive control treated sites and were significantly different from the vehicle and untreated sites. This minimally invasive methodology enabled a quantitative evaluation of potent antioxidant activity in situ in the *stratum corneum* reflecting real‐life skin conditions and confirming the benefits of the topical application of a product containing 3 antioxidants in the prevention of UVA‐induced oxidative damage.

## 1 INTRODUCTION

Skin ageing is caused by the combination of intrinsic and extrinsic factors leading to the continuous formation of reactive oxygen species (ROS),^1^ which induce physiological and structural changes in the skin. UV radiation, potent generator of skin ROS, can upset the balance between pro‐oxidants production and antioxidant defense and can therefore directly promote oxidative DNA damage and the peroxidation of lipids and proteins in the skin.^2^ This contributes to related oxidative stress skin conditions, including photoallergy,^3^ photoageing^4^ and photocarcinogenesis.^5^

The type of ROS generated depends on the UV wavelength: UVB mainly stimulates the production of $O_{2}^{-}$ through the activation of NADPH oxidase and respiratory chain reactions, while UVA produces ^1^O_2_ through a photosensitizing reaction with internal chromophores such as riboflavin and porphyrin. UVA also generates $O_{2}^{-}$ through NADPH oxidase activation^6^ and the photosensitization of advanced glycation products.^7^ The chronological ageing process as well as UV stress can deplete the cutaneous antioxidant (AO) system, leading to oxidative stress that ultimately induces oxidative damage to proteins, lipids and nucleic acids in skin cells.^8^

Antioxidant molecules in the skin interact with ROS or their by‐products to either eliminate the ROS or minimize their deleterious effects. These antioxidant molecules include glutathione (GSH), α‐tocopherol (vitamin E), ascorbic acid (vitamin C), GSH peroxidases, GSH reductase, GSH S‐transferases, superoxide dismutases (SODs), catalase and quinone reductase.^8^, ^9^

A common strategy to prevent skin ageing is to exogenously supplement the skin with an association of several antioxidants.^10^, ^11^ These include common vitamins such as ascorbic acid, tocopherol, coenzyme Q and their derivatives. Additionally, to ‘boost’ antioxidant systems within the skin, activators of several enzyme systems that regenerate these antioxidants, such as GSH peroxidases, GSH reductase, GSTs or SODs, catalase and quinone reductase) are also used topically. Tsai et al^12^ evaluated AO activity in the *stratum corneum* (SC) of the human forehead for seven AOs commonly used in cosmetic products using the DPPH scavenging assay. They found that α‐tocopherol and ascorbic acid had the strongest antioxidative capacities. The antioxidative activity of AOs in human SC proteins through the topical supplementation of antioxidants has been demonstrated to provide additional protection in neutralizing ROS from both endogenous and exogenous sources.^13^

Alpha‐tocopherol (major lipophilic endogenous AO) is used in many cosmetic formulations and represents the predominant SC antioxidant with respect to its concentration and its unique susceptibility to the various oxidative challenges tested.^14^ However, in its natural form, it is photo‐unstable and is almost completely depleted after 15 minutes of solar‐simulated UV irradiation.^15^

The photo‐instability and short duration of tocopherol in the skin can be addressed by combining tocopherol and a glucoside (delta‐tocopherol glucoside, delta‐TG or pre‐tocopheryl^®^). This combination is a prodrug that allows skin delivery using the extracellular enzyme glucosidase, which is involved in maintaining epidermal barrier function.^16^

As reported by Mavon et al^17^ under the different test conditions (ie two skin models, two concentrations, three test times and compartmental analysis), delta‐tocopherol glucoside was metabolized into free tocopherol. In the reconstituted human epidermis, over 90% of the delta‐tocopherol glucoside was bioconverted after 18 hours. The association of retinaldehyde (RAL) with delta‐tocopherol glucoside has been proven to be more effective than RAL alone on a viable human skin model submitted to UVA and UVB irradiations.^18^, ^19^

Glycylglycine ole‐amide (GGO) is a small amphiphilic molecule intended to protect the connective tissue of the skin from glycation and elastosis, with a demonstrated effect on UV‐induced protein glycation in the dermis.^20^, ^21^ In the human keratinocyte cell line A431, GGO potentiated the esterification of retinol following retinaldehyde application.^22^

Free radicals in the skin can be generated by UV irradiation or chronic damage. The most widely used methods for assessing the antioxidant/radical scavenging activity of a compound are in vitro methods involving hydrogen atom transfer (HAT) and electron transfer (ET)‐based assays.^23^ Nevertheless, in vitro methods do not reflect the actual biological processes in skin cells, and results obtained with cell lines, although similar, should be interpreted with caution. In vivo methods have the advantage of reflecting real‐life conditions but are invasive. SC stripping is a commonly used ex vivo method for evaluating skin kinetics and the penetration depth of topical products^23^ and for evaluating lipid peroxidation by natural skin AOs and cosmetics.^24^ This validated method was used by Alonso et al^25^ to evaluate an emulsion containing α‐tocopherol, ascorbic acid and resveratrol compared to an emulsion containing fish extract. This method allowed the degree of SC lipid peroxidation inhibition to be determined and thus, indirectly, the antioxidant property of the cosmetics. The reliability of this method, combined with its minimally invasive procedure, is also supported by the key role of SC in skin AO potential and reflects the skin's AO reservoir. Skin stripping has also been used to evaluate transcriptomic, inflammation markers and skin lipids.^26^, ^27^, ^28^

This randomized intra‐individual active‐ and vehicle‐controlled clinical study aimed to evaluate the capacity of a topical product containing 0.05% RAL, 0.1% delta‐tocopherol glucoside (delta‐tocopherol glucoside^®^) and 0.1% GGO to increase the skin AO pool and its in situ antioxidant activity after UVA irradiation compared with a 2% alpha‐tocopherol cream, a vehicle and a non‐treated site using a reliable ex vivo analytical method.

We describe here one exploratory in vivo study performed to assess the potential effect of a cosmetic cream containing RAL, GGO and pre‐tocopheryl in adult healthy volunteers.

## 2 METHODS

### 2.1 Subjects

In this monocentric randomized, placebo‐ and active‐controlled, intra‐individual comparison, exploratory study, twenty healthy Caucasian females and males were included by a board‐certified dermatologist. Random allocation and a positive and a negative control were included in the study protocol. All of the study procedures were carried out according to the World Medical Association (WMA) Helsinki Declaration and its amendments (Ethical Principles for Medical Research Involving Human Subjects, adopted by the 18th WMA General Assembly Helsinki, Finland, June 1964).^29^, ^30^ All subjects provided written informed consent prior to participation in the trial (ID ISRCTN49855247).

Eligible participants were all adult females and males aged between 18 and 70 years (39.1 ± 10.1 years) with clinical signs of skin ageing and Fitzpatrick skin types II and III (Table S1). Subjects were healthy volunteers. Exclusion criteria were pregnancy or intention to become pregnant, lactation, allergy/sensitivity to cosmetics, pharmacological treatment with photosensitizing drugs or with drugs/food supplements able to induce skin colouring, unwillingness or inability to comply with the requirement and constraints of the study protocol, use of topical products or food supplements containing antioxidant ingredients, exposition to sun two months before the start of the study and regular use of tanning beds. Throughout the study period, the subjects were asked to avoid both natural and artificial UV exposure.

The study took place at the Farcoderm (now Complife Italia) dermatological facilities in San Martino Siccomario, Pavia, Italy, from April to June 2012. Farcoderm is an independent testing laboratory working with the University of Pavia which specializes in the in vitro and in vivo safety and efficacy assessment of cosmetics, food supplements and medical devices.

### 2.2 Treatment procedure

#### 2.2.1 Products

The tested product was a facial skin care dermo‐cosmetic (Ysthéal, Avène, Pierre Fabre Dermo‐Cosmétique) containing retinaldehyde 0.1%, pre‐tocopheryl^®^ 0.1% and GGO 0.1% and Avène spring water (Avène Aqua) in a cream base. The active control was a cream base with 2% alpha‐tocopherol, and the placebo/vehicle control was cream base only without any active ingredients.

#### 2.2.2 Applications

**Figure 1:**
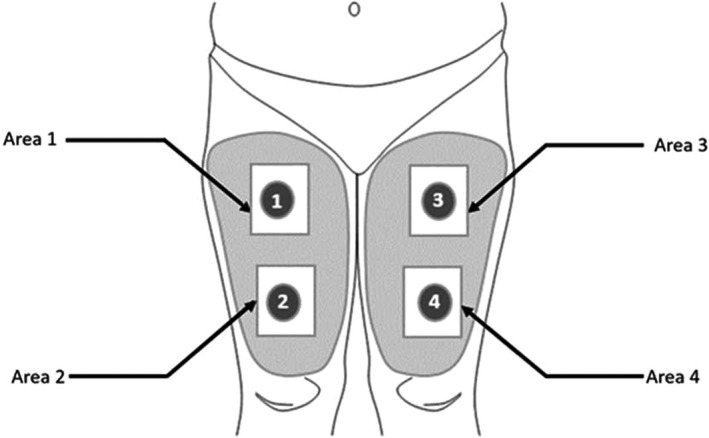
Study design. The product to be tested and benchmarks were applied on the upper/lower part of the right/left leg according to the randomization diagram. Four areas were defined: one for the tested product, one for the vehicle control, one for the active control and the last one without any product (untreated zone)

After enrolment, four skin sites on the right and left legs (Figure 1) were randomly allocated for a double‐blind treatment with the test product, active control or placebo cream, the fourth site remaining untreated. For allocation, a 4 × 4 array (Latin square) was completed for each test condition. The rows of the Latin square were then randomly replicated five times. Subjects attended visits at clinic at baseline and after 15 and 30 days of treatment. At each visit, subjects were exposed to UVA irradiation. Skin strippings were collected on non‐exposed treated and control skin sites, as well as on the UVA‐exposed treated and control skin sites (4 and 24 hours after UVA exposure).

#### 2.2.3 Description of skin stripping

Non‐invasive skin sampling was carried out using Corneofix^®^ (Courage and Khazaka, Electronics GmbH) adhesive foils. At baseline, after 15 and 30 days of treatment and 4 hours after UVA exposure (at each experiment time), 10 skin strippings were collected under standard pressure conditions. The first stripping was discarded, while stripping numbers 2, 3 and 10 were collected and stored at −80°C for biochemical assays.

#### 2.2.4 UVA exposure

A 1‐cm^2^ skin site inside each leg was exposed to 5 J/cm^2^ UVA dose for 2 minutes using a multiport 601 solar simulator (Solar^®^ Light Company). The output of UVA radiation was controlled using a UVA Detector PMA 2113 LLG (Solar^®^ Light Company) connected to a PMA2100 radiometer (Solar^®^ Light Company). The dose rate was 2.5 J/cm^2^/min, which is equivalent to 41.7 mJ/cm^2^/s.

#### 2.2.5 Skin antioxidant activity: FRAP assay

Skin antioxidant activity was measured on the first two skin layers using the ferric reducing ability of a plasma (FRAP) assay, as described by Benzie and Strain^31^ with minor modifications. FRAP is based on the reduction of a ferric‐tripyridyltriazine (FeIII‐TPTZ) complex to the ferrous (FeII) form at a low pH. The ferric to ferrous reduction causes a blue‐coloured ferrous‐tripyridyltriazine complex to form. Briefly, 100 µL of distilled water and 400 µL of working FRAP reagent (25 mL acetate buffer, 2.5 mL TPTZ solution and 2.5 mL FeCl_3_ 6H_2_O solution) were added to 12‐multiwell plates containing the strips. Adhesive foils were placed in the 12‐multiwell plate with the adhesive part (containing the SC material) high (in order to facilitate the contact between reagents and SC antioxidants). The samples were then incubated at 37°C using a microplate incubator/shaker with 30 minutes of continuous agitation. Absorbance was read at 595 nm.

#### 2.2.6 Skin lipid peroxidation induced by UVA radiation: MDA test

Baseline and UVA‐induced skin lipid peroxidation (LPO) were measured on the 10 skin layer using the malonyldialdehyde (MDA) assay as described by Eldermeier et al^32^ in 1998 with minor modifications. The MDA assay is based on the ability of the chromogen *N*‐methyl‐2‐phenylindole (NMPI) to react with MDA at 45°C under acidic conditions to produce a stable chromophore that has an absorption peak at 586 nm. Each well of the 12‐multiwell plates was filled with 500 µL of a 0.5 mmol/L CuSO_4_ aqueous solution and incubated at 37°C using a microplate incubator/shaker with 1 hour of continuous agitation. After incubation, 1.3 mL R1 solution (2.13 mg *N*‐methyl‐2‐phenylindole/mL acetonitrile) and 0.3 mL 37% HCl were added, and the samples were further incubated at 45°C for 60 minutes under continuous agitation. The reaction was stopped with the use of ice for 10 minutes followed by 10 minutes at room temperature. 1 mL of solution was centrifuged at 120*g* for 10 minutes. Absorbance was read at 586 nm.

#### 2.2.7 Statistical analysis

Statistical analysis was carried out on the intention‐to‐treat population using NCSS 8 (version 8.0.4 for Windows; NCSS, LLC). Data normality was verified using skewness, kurtosis and Shapiro‐Wilk *W* tests at *α* = .05. Departure from normality was observed in the data set; thus, a two‐tailed Wilcoxon signed rank test for difference in medians was used for both intra‐ and inter‐group comparisons. The statistical significance probability value was set at *P* < .05. Values are reported as mean ± standard error of mean (SEM).

## 3 RESULTS

All twenty volunteers completed the study. There were no dropouts or missing results.

### 3.1 Effect on the skin antioxidant pool

**Figure 2:**
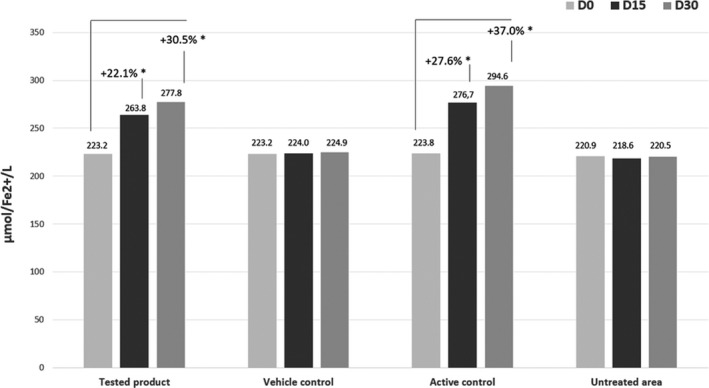
Mean skin antioxidant pool (FRAP test) µmol/L Fe^2+^ according to the randomization group. The skin antioxidant pool has been measured compared to baseline (D0) after 15 (D15) and 30 d (D30) of daily application of the tested product. *Indicates a significant difference from D0 (Wilcoxon signed rank test, *P* < .05)

The antioxidant activity of the tested product was measured by comparing the FRAP results before and after cumulative topical application of the tested product on treated vs control sites. FRAP is a direct measurement of the total reductive power of the biological matrix and an indirect index of the capacity of the considered system to resist oxidative damage. Data on skin antioxidant power are reported in Figure 2.

After 15 days of application, the levels of skin AO were significantly higher in the treated group than in the vehicle group (*P* < .001) with an increase of 22.1% from baseline. This increase was consistent with the results observed on the active control site (27.6% increase, *P* < .001).

No statistically significant differences were observed between baseline and D15 values on the vehicle control site (*P* = .69) or on the untreated site (*P* = .61).

After 30 days of daily application of the dermo‐cosmetic product, the skin AO pool significantly increased by 30.5% (*P* < .001). With the alpha‐tocopherol 2% active control, the skin AO pool increased by 37% (*P* < .001). No statistically significant differences were observed between the baseline and D30 values on the vehicle control site (*P* = .41) nor on the untreated site (*P* = .75). Differences in skin AO pool improvement between the tested product and active control treated sites were not statistically significant at day 15 (*P* = .046) or at day 30 (*P* = .03, Wilcoxon signed rank test for median differences).

### 3.2 Effect on skin lipoperoxidation before UVA exposure

Lipid peroxidation was measured using the MDA test concentration of malonyldialdehyde (MDA) and 4‐hydroxynomenal, the two main products of LPO, which is a good indicator of lipoperoxide damage in a biological system. The MDA level in each group was assessed at baseline, D15 and D30. The corresponding data are presented in Table 1.

**Table 1:** Mean data for lipoperoxidation values (µmol/L, mean ± SEM) and % change from baseline

	Tested product	Vehicle	Active control	Untreated area
LPO D0	3.02 ± 0.23	3.10 ± 0.25	3.06 ± 0.23	3.09 ± 0.24
LPO D15	2.49 ± 0.13	3.15 ± 0.24	2.56 ± 0.13	3.09 ± 0.24
Variation (%) D15 vs D0	−13.30%*	+3.30%	−13.60%*	+0.40%
LPO Day 30	2.46 ± 0.10	3.35 ± 0.23	2.55 ± 0.14	3.23 ± 0.22
Variation D30 vs D0	−11.50%*	+8.50%	−12.10%*	+7.20%

Abbreviations: D0, day 0; D15, day 15; D30, day 30; LP0, lipid peroxidation; SEM, standard error of mean.

* *P* < .05: statistical analysis by Wilcoxon signed rank test.

At day 15, a statistically significant decrease in skin MDA content of −13.3% (*P* < .001) and of −13.6% (*P* < .001) was measured on the tested product and active control treated sites, respectively. The skin MDA content remained unchanged both on the vehicle control site (*P* = .695) and on the untreated site (*P* = .837).

At day 30, a statistically significant decrease in skin MDA content of −11.5% (*P* < .001) and of −12.1% (*P* < .001) was observed on the tested product and active control treated sites, respectively. The skin MDA content remained unchanged both on the vehicle control site (*P* = .271) and on the untreated site (*P* = .218).

Differences in the skin MDA content between the tested product and active control treated sites were not statistically significant either at day 15 or at day 30.

### 3.3 Effect on post‐UVA exposure value of skin lipoperoxidation

**Figure 3:**
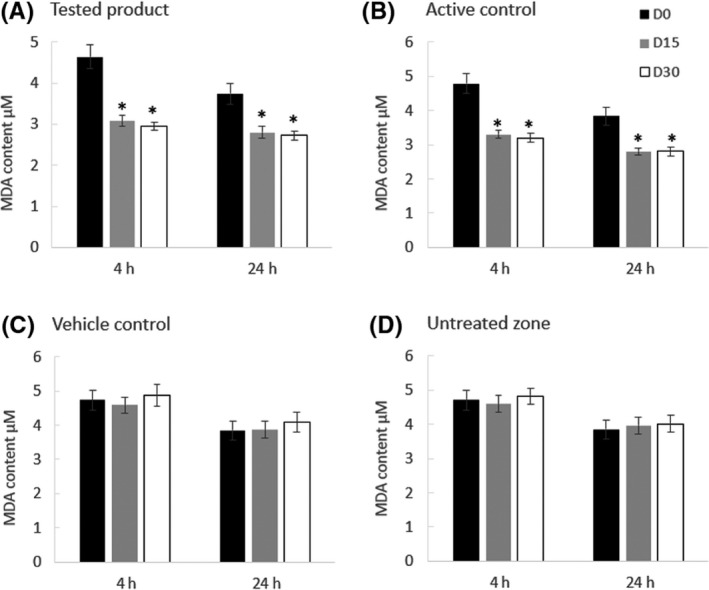
Skin lipoperoxidation (MDA test results) after UVA exposure. Skin lipoperoxidation was assessed in the skin strippings after 15 and 30 d of daily application of the tested product (A) containing antioxidants, the active control (B) and the vehicle control (C) 4 and 24 h after UVA radiation. The skin lipoperoxidation levels measured in the untreated site are presented in D. Abbreviations: D0, day 0; D15, day 15; D30, day 30.*Significant decrease from D0 (Wilcoxon signed rank test, *P* < .05)

The MDA test was performed on the skin stripping collected after UVA exposure. Data on UVA‐induced skin lipoperoxidation are reported in Figure 3.

After 15 days and 30 days of the tested product application, the content of MDA decreased significantly both after 4 and 24 hours post‐UVA exposure (Figure 3A). Comparable results were obtained for the active control (Figure 3B) showing a significant decrease in MDA. No statistically significant variation was measured in the vehicle‐treated site 4 and 24 hours after the UVA exposure (Figure 3C), nor in the untreated site (Figure 3D).

After 15 and 30 days of treatment, skin LPO values were significantly (*P* < .001) lower on the tested product and active control sites compared to the vehicle control and untreated sites.

These results demonstrate the efficacy of the tested product to replenish the skin with antioxidants, to improve the skin capacity to oppose oxidative stress and to decrease the peroxidation of skin membrane lipids after UVA exposure.

## 4 DISCUSSION

The cutaneous antioxidant system is complex and far from being completely understood. Thiele et al^33^ demonstrated that α‐tocopherol is, according to the respective levels in the epidermis, the major antioxidant in the human SC. Its depletion is a very early and sensitive biomarker of environmentally induced oxidation. A physiological mechanism enables the transport of α‐tocopherol to the skin surface *via* sebaceous gland secretion. There is conclusive evidence that the levels of protein oxidation increase towards the outer SC layers and antioxidants contained in the SC, particularly vitamin E, are more susceptible to UV radiation due to a lack of co‐antioxidants in this outermost skin layer. The direct depletion of α‐tocopherol and the formation of free radicals also affect other endogenous antioxidant pools in a manner similar to a chain‐breaking antioxidant activity.^10^

It is now well known that the SC has a real and effective antioxidant network (both enzymatic and non‐enzymatic) acting as a very early and sensitive biomarker of environmentally induced oxidation.^33^ Besides the recognized physical barrier properties, *SC* has also photoprotective properties^33^, ^34^ due to both physical and biochemical mechanisms. Under environmentally challenging conditions, topical application of antioxidants could support physiological mechanisms to maintain or restore a healthy skin barrier and to prevent UV‐induced photoageing.

Cosmetics, by definition, cannot have any ‘active’ impact on the skin physiology nor significant penetration: they can only work only in the outermost layer of the skin (*SC*).^35^

Over the last decade, antioxidants have been used as functional ingredients for anti‐ageing preparations to prevent and modulate oxidative skin damage. Up to this point, no model with the exception of photo‐induced oxidative in vitro skin damage has shown sufficient reproducibility to validate the above‐stated claim in a finished cosmetic product. Actually, in vitro methods do not reflect the actual biological processes of skin cells, and the results obtained with cell lines, although similar, are to be interpreted with caution.

In vivo methods present the advantage of reflecting real‐life conditions but are invasive and thus cause some impairment to study subjects. To this end, we developed an in situ protocol based on a ROS‐driven micro‐inflammatory model of oxidative skin damage in stripped skin. The measurements were carried out both in the uppermost (FRAP) and in the lowest part (MDA) of the SC. Regarding the study design, our aim was to investigate the supportive effect of exogenous antioxidants application on stratum corneum antioxidant network as a first barrier to prevent the pathophysiological process (due to environmental stressor) occurring deeply in the epidermis and in the dermis, and the protective effect of exogenous antioxidant application in preventing the SC antioxidant depletion due to UVA. Based on the supportive evidences and the study aim, the study design met the study needs.

Our in situ study consisted of generating reactive oxygen species by UVA‐induced micro‐inflammation; the relationship between skin ageing and inflammation is at the basis of the micro‐inflammatory model of skin ageing.^36^, ^37^ This suggests that the peroxidation of skin cellular lipids induced by endogenously or exogenously generated free radicals represents the promoter of a subclinical inflammatory state. Our working hypothesis was that exposure to radiation induces surface molecular modifications such as lipid peroxidation. These modifications are known to trigger the peroxidative cascade and are likely to damage skin cells, thus activating the micro‐inflammatory reaction. The micro‐inflammatory model of skin ageing challenging experiments described by Giacomini et al^37^ indicates that surface oxidative damage can be generated in symmetric anatomical sites but can be hindered in a controlled manner by the appropriate use of topically applied antioxidants. The application of antioxidants on one leg site but not on the other for individuals in a cohort allows an analysis of the effects (if any) of molecular surface damage. By allowing smaller amounts of surface peroxides to be formed, we would expect antioxidants to reduce the rate of modification of the macroscopic properties of the skin, such as thickness and elasticity.

UVA (320‐400 nm) is the predominant UV band reaching the earth's surface. UVA penetrates the skin deeper and induces effects on both the epidermal and dermal compartments. The biological effects induced by UVA are mainly related to ROS generation.^38^ There is now strong evidence that UVA contributes to the development of skin cancers^39^, ^40^ by impairing the phenotypic and functional maturation of human dendritic cells into potent antigen‐presenting cells.^41^

In our protocol, due to ethical principles, we chose to prevent UVB‐induced erythema by restricting the skin exposure to the UVA wavelengths. The dose of UVA that we used can be considered as physiologically relevant because approximately 5 J per cm^2^ UVA was received after about 2 minutes of exposure to a solar lamp at a dose rate of 2.5 J/cm^2^. The UVA dose was chosen based on preliminary research carried out before this study, and the MDA measurements were considered robust and representative of AO activity without inducing erythema or phototoxic reaction.

The SC skin stripping technique has been previously described to assess antioxidant enzymes and adapted to the evaluation of reducing antioxidant capacity in skin samples.^37^, ^42^ In the present study, free radical scavenging activity was determined by the FRAP method. The difficulty in measuring each antioxidant component separately as well as their interactions required quick, simple and efficient assays, which used different principles to measure antioxidant capacity.^43^ The FRAP method was chosen to assess in a reliable and reproducible method the total antioxidant capacity of SC and to reflect the antioxidant pool present in the cutaneous antioxidant system. This reliable method was previously described to measure the effects of nutraceutical or topical antioxidant on the extent of the skin antioxidant pool.^44^

The results of this study showed that the topical application of a product containing RAL, delta‐tocopherol glucoside and GGO for 15 consecutive days increased the skin AO pool and significantly reduced the oxidative damage caused by UVA irradiation 4 and 24 hours postdose compared to the vehicle or untreated sites.

There was no difference in AO activity between the tested product and the positive control (2% alpha‐tocopherol). Notably, the concentration of tocopherol in the positive control was 20‐fold higher than the delta‐tocopherol glucoside concentration (0.1%) in the tested product. One can conclude that lower concentrations of tocopherol in the tested product combined with GGO and retinaldehyde had an AO effect similar to that of the active 2% alpha‐tocopherol control. This could be explained by synergistic interactions that contribute to the AO pool and to UVA‐induced oxidative damage reduction because these in situ results are consistent with previous in vitro studies of long‐term viable human skin culture after UV damage reporting the anti‐ageing efficacy of 0.05% RAL in association with a delta‐tocopherol glucoside formulation.^21^ An in vitro study on senescent fibroblasts demonstrated the anti‐ageing properties of an association of RAL, delta‐tocopherol glucoside and GGO by acting on different mechanisms involved in skin ageing. Indeed, RAL is able to increase proteasomal activity and methionine sulfoxide reductase A to reverse the effects of oxidative damage.^21^ RAL is also able to increase type I collagen and decrease MMP‐1 synthesis. OGG is a powerful inhibitor of glycation, and delta‐tocopherol glucoside is a strong antioxidant.^45^ Furthermore, protein damage mediated by oxidation, such as advanced glycated end products and products of lipid peroxidation, is implicated during skin ageing. The addition of GGO has a proven effect on protein glycation in the dermis due to UV. In vitro study of senescent fibroblasts (Hayflick model) demonstrated anti‐glycation properties of GGO on human skin explants and showed that GGO is able to prevent the inhibitory effect of methylglyoxal (a strong glycation inducer) on fibrillin‐1 expression. Another study demonstrated a substantial benefit of the same dermo‐cosmetic containing RAL, delta‐tocopherol glucoside and GGO in treating signs of ageing.^46^

Our methodology, while minimally invasive, allowed the ‘in situ’ evaluation of the AO and reflected real‐life skin conditions. These results suggest that daily use of the dermo‐cosmetic containing RAL, GGO and delta‐tocopherol glucoside enhances resistance to oxidative stress and may help prevent skin ageing by blocking oxidative effects.

## CONFLICT OF INTEREST

Virginie Ribet is an employee of Pierre Fabre Dermo‐Cosmétique. Ana Beatris Rossi was an employee of Pierre Fabre Dermo‐Cosmétique at the time of the study. Doctor Vincenzo Nobile from Farcoderm, member of the Complife group, Pavia, received financial compensation from Pierre Fabre Dermo‐Cosmétique for conducting the study.

## AUTHOR CONTRIBUTION

V. R. contributed to the concept and design of the study (main protocol author), the implementation and follow‐up of the study, and the analysis and interpretation of data. V. N. contributed to the concept and design of the study (medical expert). V. N. had full access to all study data and takes responsibility for data integrity and the accuracy of the data analysis. V. N. performed data analysis and supervised the entire reviewing process. A. B. R. participated in blinded review, analysis and interpretation of the data and provided medical expertise. V. R., A. B. R. and V. N. reviewed and critically revised the manuscript and gave final approval of the version to be published.

## Supporting information


**Table S1.** Subject demographic information

**Table S2.** Skin LPO levels in each experimental condition
