# Supplementary material for: In situ antioxidant activity of a dermo‐cosmetic product: A randomized controlled clinical study
Source: Exp Dermatol. 2019 Sep 30;28(11):1219–26. doi: 10.1111/exd.14005 (PMC6973136; doi:10.1111/exd.14005)
Supplement: Supplementary file 1 — Table S1. Subject demographic information [file EXD-28-1219-s001.docx]

**Table S1.** Subject demographic information

| **Subjects demography** | |
| --- | --- |
| Age (mean ± SEM) | 39.1 ± 10.1 |
| Minimum age | 25 |
| Maximum age | 54 |
|  |  |
| Sex |  |
| Female | 85% |
| Male | 15% |
| Skin type† |  |
| Phototype II | 45% |
| Phototype III | 45% |
| Phototype IV | 10% |

Abbreviations: SEM: standard error of mean

† according to Fitzpatrick classification
