# Supplementary material for: In situ antioxidant activity of a dermo‐cosmetic product: A randomized controlled clinical study
Source: Exp Dermatol. 2019 Sep 30;28(11):1219–26. doi: 10.1111/exd.14005 (PMC6973136; doi:10.1111/exd.14005)
Supplement: Supplementary file 2 — Table S2. Skin LPO levels in each experimental condition [file EXD-28-1219-s002.docx]

**Table S2.** Skin LPO levels in each experimental condition and variation compared to baseline levels.

|  | Raw data (µM, mean± SEM) | | | Variation (%) vs T 0 h | |
| --- | --- | --- | --- | --- | --- |
| D0 | T0h | T4h | T24h | T4h | T24h |
| Tested product | 3.02±0.23 | 4.64±0.29 | 3.74±0.26 | 57.5% * | 26.7% * |
| Vehicle control | 3.10±0.25 | 4.73±0.30 | 3.84±0.28 | 57.0% * | 25.9% * |
| Active control | 3.06±0.23 | 4.78±0.29 | 3.83±0.26 | 60.2% * | 27.5% * |
| Untreated area | 3.09±0.24 | 4.70±0.29 | 3.85±0.29 | 56.2% * | 26.0% * |
| D15 |  |  |  |  |  |
| Tested product | 2.49±0.13 | 3.08±0.14 | 2.80±0.13 | 25.4% * | 13.5% * |
| Vehicle control | 3.15±0.24 | 4.58±0.23 | 3.86±0.25 | 51.2% * | 24.9% * |
| Active control | 2.56±0.13 | 3.30±0.12 | 2.80±0.10 | 31.7% * | 11.4% * |
| Untreated area | 3.09±0.24 | 4.59±0.25 | 3.97±0.25 | 53.4% * | 31.7% * |
| D30 |  |  |  |  |  |
| Tested product | 2.46±0.10 | 2.95±0.09 | 2.72±0.10 | 21.3% * | 11.0% * |
| Vehicle control | 3.35±0.23 | 4.87±0.33 | 4.08±0.29 | 46.3% * | 21.7% * |
| Active control | 2.55±0.13 | 3.20±0.14 | 2.80±0.13 | 27.3% * | 10.6% * |
| Untreated area | 3.23±0.22 | 4.81±0.24 | 4.01±0.25 | 52.9% * | 25.8% * |

Abbreviations: LP0, lipid peroxidation; SEM: standard error of mean; D0: Day 0; D15: Day 15; D30: Day 30; T0h: baseline; T4h: 4 hours after baseline measure, T24h: 24 hours after baseline measure.

*p<0.05: statistical analysis by Wilcoxon signed rank test
